# Assessment of Health-Related Quality of Life in Gastric Cancer Patients: A Cross-Sectional Study in North-Eastern Kazakhstan

**DOI:** 10.3390/healthcare14193170

**Published:** 2026-09-24

**Authors:** Zhamilya B. Kassymbay, Saulesh A. Apbassova, Arnakyzy Yerkezhan, Assel R. Tukinova, Gulnar M. Shalgumbayeva

**Affiliations:** 1 Department of Pathological Anatomy and Forensic Medicine, NJSC “Semey Medical University”, Semey 071400, Kazakhstan; 2 Department of Epidemiology and Biostatistics, NJSC “Semey Medical University”, Semey 071400, Kazakhstan; 3 Department of General Medical Practice of Semey City, NJSC “Semey Medical University”, Semey 071400, Kazakhstan

**Keywords:** gastric cancer, health-related quality of life, EORTC QLQ-C30, EORTC QLQ-STO22, Kazakhstan

## Abstract

**Background/Objectives:** Gastric cancer severely affects health-related quality of life (HRQoL). This study evaluated HRQoL profiles and identified clinical, functional, and symptom correlates among gastric cancer patients in Kazakhstan. **Methods:** A cross-sectional study was conducted in a single-center cohort (*n* = 387) using validated Russian-language European Organisation for Research and Treatment of Cancer Quality of Life Questionnaire-Core 30 (EORTC QLQ-C30) (Kazakh verbal clarification provided when needed, *n* = 12). Hierarchical multivariable linear regression was performed to identify factors independently associated with Global Health Status/QoL (GHS). **Results:** Mean GHS score was 51.9 ± 24.3. Major reported symptom burdens included stomach area pain, fatigue, and anxiety. In the final adjusted regression model (Step 3), GHS was significantly predicted by older age (ß = −0.21, *p* < 0.001), pain (ß = −0.37, *p* < 0.001), body image (ß = −0.12, *p* = 0.035), and functional domains: physical (ß = −0.20, *p* = 0.022), role (ß = 0.14, *p* = 0.026), emotional (ß = 0.32, *p* < 0.001), and cognitive functioning (ß = −0.14, *p* = 0.018). Employment categories also remained significant predictors: white-collar (ß = 0.09, *p* = 0.044), service sector (ß = 0.20, *p* < 0.001), blue-collar (ß = 0.19, *p* < 0.001), and retired (ß = 0.16, *p* = 0.022). Anxiety showed a positive association with GHS (ß = 0.24, *p* < 0.001), reflecting a statistical suppression effect, whereas fatigue (ß = −0.02, *p* = 0.745) was not significant after controlling for covariates. **Conclusions:** HRQoL in gastric cancer patients is strongly associated with physical symptom burden, age, and functional status. Targeted pain management, psychological support, and holistic care should be prioritized in routine oncology practice.

## 1. Introduction

Gastric cancer (GC) remains a major global public health challenge, ranking as the fifth most common malignancy and the fifth leading cause of cancer mortality worldwide [1]. Epidemiological data from 2022 highlights this burden, with over 968,000 newly diagnosed cases and nearly 660,000 deaths reported globally [1]. Gastric cancer represents the primary oncological diagnosis and the leading cause of cancer-related mortality among men in several South-Central Asian nations, including Afghanistan, Iran, Kyrgyzstan, and Tajikistan (where it is also the primary cause of cancer death among women). Regionally, the highest incidence is observed in Eastern Asia—led globally by Mongolia across both sexes—and Eastern Europe, whereas the lowest rates are recorded throughout Africa [1].

In Kazakhstan, the burden of gastric cancer remains persistently high. Annual registered cases remained stable between 2012 (2644 cases) and 2022 (peaking at 2989 cases), with a brief decline in 2020 (2656 cases) directly attributable to COVID-19-related healthcare disruptions [2]. Between 2009 and 2018, a total of 19,672 GC deaths were recorded nationwide, with men accounting for the majority of fatalities (64.2%, *n* = 12,638) compared to women (35.8%, *n* = 7034) [3].

The development of GC is multifactorial, involving a complex interplay of genetic susceptibility and environmental factors [4]. Chronic Helicobacter pylori infection serves as the predominant etiological trigger [5,6]. Additionally, lifestyle and dietary factors significantly elevate risk; specifically, tobacco smoking, alcohol consumption, high intake of salt-preserved foods, processed meats, and grilled proteins—combined with inadequate fruit consumption—are strongly associated with increased disease susceptibility [7]. Over the past five decades, a consistent global decline in overall stomach cancer incidence has been observed, largely driven by improvements in food preservation techniques and decreasing global prevalence of H. pylori [1]. Conversely, recent epidemiological shifts reveal an increasing incidence among younger cohorts and low-risk populations, particularly for tumors near the esophagogastric junction—a trend hypothesized to be driven by rising rates of autoimmune gastritis and gastric microbiome dysbiosis [8,9].

While traditional survival and cure rates remain fundamental clinical endpoints, they provide a limited perspective on the comprehensive physical, psychological, and functional burden experienced by survivors. Consequently, Health-Related Quality of Life (HRQoL) has evolved into a cornerstone patient-reported outcome measure [10]. HRQoL evaluates how therapeutic strategies impact a survivor’s functional recovery within the context of their individual expectations, cultural norms, and personal values [11]. It encompasses physical, mental, emotional, social, and sexual functioning [12], directly aligning with the World Health Organization’s global cancer control strategy to prioritize QoL enhancement [13].

Evidence demonstrates that GC and its therapeutic interventions severely compromise patient QoL due to physiological burdens like nausea and dysphagia [14], psychological distress such as depression [15], and social withdrawal driven by illness-related stigma [16,17]. Therefore, assessing HRQoL is imperative for monitoring long-term treatment sequelae and optimizing supportive care. However, data evaluating HRQoL and its specific correlates in Central Asian populations remain limited. To address this gap, this study aimed to evaluate HRQoL and identify its key associated factors among patients with gastric cancer in the North-Eastern region of Kazakhstan using validated European Organisation for Research and Treatment of Cancer Quality of Life Questionnaire-Core 30 (EORTC QLQ-C30) and Gastric Cancer Module (EORTC QLQ-STO22).

## 2. Materials and Methods

### 2.1. Study Design and Participants

This cross-sectional study was conducted in the North-Eastern region of Kazakhstan from January to March 2025. The study enrolled respondents permanently residing in the North-Eastern region of Kazakhstan (Pavlodar and East Kazakhstan regions, and the Abai region). This geographical choice is fundamentally driven by the high epidemiological burden of gastrointestinal malignancies in North-Eastern Kazakhstan and the need to evaluate patient-reported outcomes in regional cancer care settings. Given the elevated incidence of gastric cancer in this population, assessing health-related quality of life provides essential insights into patients’ psycho-emotional and physical status, helping to identify unmet supportive care needs in regional oncology centers.

Due to practical constraints in accessing the full regional cancer registry frame, a consecutive convenience sampling strategy was employed across participating regional oncology centers. To determine statistical adequacy, sample size requirements were evaluated for multivariable modeling rather than general population estimates. For a hierarchical multiple linear regression model containing up to 22 parameters, standard sample size guidelines (e.g., Green’s rule: *n* > 50 + 8 m = 226, where m is the number of predictors) were fully satisfied. A formal a priori power analysis (noncentral F-distribution, α = 0.05, k = 22 predictors) confirmed that our sample of *n* = 387 provides power exceeding 99% to detect medium effect sizes (f^2^ = 0.15) in the overall model. Power to detect smaller effects was more limited (93% for f^2^ = 0.08; 28% for f^2^ = 0.02), a consideration relevant to interpreting non-significant individual predictors in the final model (e.g., TNM stage, gender, residency). A total of 401 questionnaires were initially collected upon written informed consent. To ensure data quality, trained research staff reviewed returned paper surveys on-site. Fourteen questionnaires were excluded from the analysis due to exceeding the pre-established threshold of >50% missing items per scale, resulting in a final analytic cohort of *n* = 387 participants (*n* = 375 independent Russian-version completers and *n* = 12 with verbal Kazakh assistance) with complete data across the evaluated parameters. For retained questionnaires containing isolated missing items within an otherwise valid scale, the standard EORTC mean-imputation scoring rule was applied, in accordance with the EORTC Scoring Manual, yielding complete scale scores for all 387 participants.

The inclusion criteria were clear consciousness; normal language expression and comprehension abilities; ability to self-report their health outcomes; age ≥ 18 years; a confirmed diagnosis of gastric cancer at any stage, and receipt of treatment at oncology centers in the three study regions; voluntary participation in the study. The exclusion criteria were refusal to participate in the study; comprehension or reading disabilities; concurrent other cancers, and non-primary gastric cancer; residence outside the study regions of Kazakhstan, the presence of cognitive impairment, and severe comorbid conditions. All questionnaires were administered in paper-and-pencil format. Participants were asked to complete the questionnaires independently, with trained research assistants present to answer any questions without influencing the responses.

The study protocol was approved by the Ethics Committee of Semey Medical University, Kazakhstan (Protocol No. 2 dated 12 December 2023).

### 2.2. Quality of Life Assessment

The QoL was assessed using validated Russian-language European Organisation for Research and Treatment of Cancer Quality of Life Questionnaire-Core 30 (EORTC QLQ-C30) and Gastric Cancer Module (QLQ-STO22). The QoL assessment was based on the answers to all 52 items, as completed by the responders themselves.

Gastric cancer predominantly affects older age groups in Kazakhstan, most of whom are traditionally Russian-speaking. Data collection was primarily conducted using the officially authorized Russian-language versions of the EORTC QLQ-C30 and EORTC QLQ-STO22 questionnaires, obtained from the official EORTC Quality of Life Group repository for academic non-commercial research. [https://qol.eortc.org/questionnaires/, accessed on 15 May 2025]. Given that the study cohort predominantly comprised older adults in North-Eastern Kazakhstan, the vast majority of participants completed the official Russian version independently. For a minor subset of bilingual patients *n* = 12 (3.1%), verbal completion support and item clarifications were provided in Kazakh by trained bilingual research assistants based on the standard items. No standalone written Kazakh translation was created or formally evaluated. A total of 401 questionnaires were initially collected. Fourteen questionnaires were excluded due to exceeding the pre-established threshold of >50% missing items per scale, yielding a final primary analytic sample of *n* = 387 (*n* = 375 independent Russian-version completers and *n* = 12 with verbal Kazakh assistance). Additionally, 15 patients participated in an initial feasibility pre-testing phase; these pilot participants were completely excluded from the final analytic sample (*n* = 387).

The EORTC QLQ-C30 consists of 30 items categorized into multi-item scales and single items, including a global health quality of life scale, five functional scales (physical, role, emotional, cognitive, and social functioning), three symptom scales (fatigue, nausea and vomiting, and pain), and six single items assessing common cancer-related symptoms (dyspnea, insomnia, appetite loss, constipation, diarrhea, and financial difficulties). The EORTC QLQ-STO22 is a 22-item supplementary module specific to gastric cancer. It evaluates disease-specific symptoms and treatment side effects across five multi-item symptom scales (dysphagia, pain, reflux, eating restrictions, and anxiety) and four single items (dry mouth, taste alterations, body image, and hair loss). For both questionnaires, responses were transformed into linear scores ranging from 0 to 100 in accordance with the official EORTC Scoring Manual. For the global health status and functional scales, higher scores indicate a higher level of functioning and a better quality of life. Conversely, for the symptom scales and single items, higher scores represent a greater severity of symptoms and increased distress [18,19].

### 2.3. Statistical Analysis

Statistical analyses were performed using SPSS software, version 20.0 (IBM Corp., Armonk, NY, USA). Continuous variables, including the EORTC QLQ-C30 and QLQ-STO22 linear scores, were expressed as means (±) standard deviations (SD). Floor and ceiling effects were evaluated by calculating the proportion of respondents scoring the lowest (0) and highest (100) possible scores, respectively, with effects exceeding the conventional threshold of 15% considered substantial.

The Shapiro–Wilk test was performed to evaluate the normality of continuous variables. Since several QoL and symptom scales deviated significantly from normal distribution (*p* < 0.05), non-parametric Spearman rank correlation matrices were constructed to investigate bivariate linear relationships (*n* = 387). Parametric multiple linear regression was retained for multivariate analysis based on the Central Limit Theorem (*n* = 387) and confirmed through residual diagnostic testing.

Employment status was dummy-coded using ‘Unemployed’ as the reference baseline category. TNM clinical stage was entered as an ordinal continuous covariate ranging from 1 (Stage I) to 4 (Stage IV). Internal consistency reliability (Cronbach’s α) was evaluated strictly for multi-item scales; single-item symptoms and functional domains were evaluated using descriptive metrics (mean, SD, floor/ceiling effects) without internal consistency estimation.

To identify the factors associated with HRQoL of overall quality of life (Global Health Status/QoL), a three-block hierarchical multiple linear regression was conducted. Categorical independent variables were operationalized prior to modeling: Employment status was recoded into four binary dummy variables (emp_1 White-collar, emp_2 Service sector, emp_3 Blue-collar, and emp_5 Retired), using unemployed individuals as the omitted reference group; Age was treated as a continuous variable (in years); and TNM stage was analyzed as an ordinal associated factors (Stages I–IV). Independent variables were entered sequentially based on theoretical relevance: Block 1 included socio-demographic factors, Block 2 included clinical and generic QoL scales (EORTC QLQ-C30), and Block 3 included disease-specific symptom scales (EORTC QLQ-STO22). All candidate predictor variables were screened based on bivariate correlation analysis with primary outcomes and prior clinical relevance. Although all symptom scales demonstrated statistically significant bivariate associations with Global Health Status/QoL, four specific scales (dyspnea, constipation, diarrhea, and financial difficulties) were selectively omitted from the final multivariate regression steps to prevent high multicollinearity and preserve model parsimony.

Regression diagnostics were systematically performed: homoscedasticity was verified, error normality was confirmed via Q-Q plots, and multicollinearity was evaluated using Tolerance and Variance Inflation Factor (VIF) metrics. Multicollinearity was ruled out for the majority of variables (VIF < 2.5), although Physical Functioning (PF) demonstrated higher collinearity starting at Step 2 (VIF = 5.37) and reaching VIF = 5.75 at Step 3. All statistical tests were two-sided, with *p*-values < 0.05 considered statistically significant.

## 3. Results

### 3.1. Sociodemographic and Clinical Characteristics

A total of 387 patients with histologically confirmed gastric cancer completed the study. The majority of participants were male (66.7%, *n* = 258) and aged 61 years or older (69.0%, *n* = 267). Most respondents resided in urban areas (81.7%, *n* = 316) and had completed secondary education (68.2%, *n* = 264). Over half of the participants were retired (53.2%, *n* = 206). Clinically, Stage II disease represented the largest subgroup (61.0%, *n* = 236), followed by Stage III (24.3%, *n* = 94), Stage I (9.3%, *n* = 36), and Stage IV (5.4%, *n* = 21). The detailed sociodemographic and clinical profile is presented in Table 1.

### 3.2. EORTC QLQ-C30 and QLQ-STO22 Score

Distributions and Reliability Descriptive statistics, floor/ceiling proportions, and Cronbach’s α values are summarized in Table 2.

Functional Scales (QLQ-C30): Social functioning yielded the highest score (78.9 ±24.1), followed by role (71.0 ± 27.7), cognitive (69.5 ± 22.4), physical (68.0 ± 26.2), and emotional functioning (62.6 ± 27.4). Global Health Status/QoL was moderate (51.9 ± 24.3). Symptom Burden: Fatigue (44.9 ± 23.8), insomnia (42.1 ± 31.0), and appetite loss (40.6 ± 29.1) were the most prominent generic symptoms. On the disease-specific QLQ-STO22 module, anxiety was the predominant distress factor (61.1 ± 30.5), followed by body image disturbance (36.6 ± 23.8) and stomach pain (28.4 ± 21.4). Psychometrics: Substantial ceiling effects (>15%) were observed for social (40.8%), role (34.6%), and cognitive functioning (20.4%). Substantial floor effects (>15%) occurred across specific symptom domains, highest in taste problems (60.5%), constipation (54.3%), dyspnea (51.7%), and nausea/vomiting (51.2%). All multi-item scales demonstrated satisfactory-to-excellent internal consistency (Cronbach’s α = 0.70–0.94).

For the EORTC QLQ-C30 functional scales, the highest mean score was observed for social functioning (78.9 ± 24.1), followed by role functioning (71.0 ± 27.7), cognitive functioning (69.5 ± 22.4), and physical functioning (68.0 ± 26.2), suggesting relatively preserved functioning in these domains. In contrast, the mean score for global health status/quality of life was moderate (51.9 ± 24.3), indicating that overall quality of life remained substantially impaired despite relatively favorable functioning scores.

Symptom scales of the QLQ-C30 fatigue showed the highest symptom burden (44.9 ± 23.8), followed by insomnia (42.1 ± 31.0), appetite loss (40.6 ± 29.1), and financial difficulties (35.7 ± 36.3). Lower mean scores were observed for nausea and vomiting (20.1 ± 24.8), constipation (19.3 ± 23.8), and diarrhea (23.9 ± 24.9), indicating that these symptoms were less prominent in the study population.

The highest symptom of the QLQ-STO22 gastric cancer-specific questionnaire revealed that the highest symptom was found for anxiety (61.1 ± 30.5). Moderate symptom levels were observed for body image (36.6 ± 23.8), reflux (28.6 ± 19.3) and stomach area pain (28.4 ± 21.4), hair loss (25.8 ± 26.4), dry mouth (25.7 ± 26.7), and dysphagia (25.5 ± 19.9).

Acceptable score distributions was defined during assessment of floor and ceiling effects. Floor and ceiling effects were evaluated against the 15% threshold. Substantial ceiling effects were observed for social functioning (40.8%), role functioning (34.6%), cognitive functioning (20.4%), and anxiety (21.2%, *n* = 82). Notably, the substantial ceiling effect for anxiety indicates that approximately one in five patients reported the maximum score on this scale, reflecting a severe emotional burden and high psychological distress in this patient population. Physical functioning demonstrated a low ceiling effect of 6.2% (*n* = 24), whereas Global Health Status/QoL exhibited a ceiling effect of 1.6% (*n* = 6) (Table 2). Substantial floor effects were present on several symptom scales, most notably taste problems (60.5%), constipation (54.3%), dyspnea (51.7%), nausea and vomiting (51.2%), and diarrhea (43.9%), indicating that a large proportion of patients reported an absence of these specific symptoms.

For reliability, Cronbach’s α-coefficient for multi-item scales ranged from 0.70 to 0.94. The highest reliability was observed for global health status/QoL (α = 0.94), physical functioning (α = 0.92), emotional functioning (α = 0.90), and role functioning (α = 0.88). The lowest, yet acceptable, reliability coefficient was found for cognitive functioning (α = 0.70). Single-item scales (such as hair loss and body image) were excluded from the reliability analysis, as internal consistency cannot be computed for single items. These findings indicate that the multi-item scales of both the EORTC QLQ-C30 and QLQ-STO22 demonstrated satisfactory to excellent internal consistency in the studied population.

### 3.3. Bivariate Correlation Analysis

Spearman rank correlation coefficients (r_s_) between EORTC subscales and Global Health Status/QoL are reported in Table 3. Global Health Status/QoL demonstrated statistically significant positive correlations with all functional domains (r_s_ = 0.33–0.56, *p* < 0.01), led by physical functioning (r_s_ = 0.56). Conversely, Global Health Status/QoL correlated negatively with generic symptoms (e.g., pain: r_s_ = −0.61; nausea/vomiting: r_s_ = −0.55; fatigue: r_s_ = −0.45; all *p* < 0.01) and disease-specific symptoms (eating restrictions: r_s_ = −0.43; reflux: r_s_ = −0.42; dysphagia: r_s_ = −0.38; anxiety: r_s_ = −0.24; all *p* < 0.01).

### 3.4. Hierarchical Multiple Linear Regression

All regression diagnostic assumptions were met: residual analyses confirmed normal distribution of errors across all three blocks, and no critical multicollinearity issues were detected. A three-step hierarchical multiple regression was executed to identify independent determinants of Global Health Status/QoL (Table 4). Multicollinearity was monitored across all regression blocks. Across all associated factors, VIF values remained below 5.0, with the exception of the Physical Functioning (PF) scale, which exceeded this threshold starting at Step 2 (VIF = 5.37, Tolerance = 0.186) and reached its maximum at Step 3 (VIF = 5.75, Tolerance = 0.174). For Step 1, the minimum Tolerance was 0.299 and the maximum VIF was 3.34 (attributable to the dummy-coded variables). These elevated VIF values for Physical Functioning reflect expected covariance with interrelated functional and symptom subscales. Consequently, individual partial regression coefficients, particularly for overlapping scales, should be interpreted with caution.

Model fit statistics for the three-step hierarchical linear regression are summarized in Table 4. In Step 1, socio-demographic and clinical characteristics accounted for 22.9% of the variance in Global Health Status/QoL (R^2^ = 0.229, F (9, 377) = 12.415, *p* < 0.001). The addition of the generic EORTC QLQ-C30 functional and symptom scales in Step 2 yielded a highly significant increase in explained variance (ΔR^2^ 0.279, F-change (10, 367) = 20.759, *p* < 0.001). Finally, adding the disease-specific EORTC QLQ-STO22 scales in Step 3 provided further statistically significant incremental variance explanatory power (ΔR^2^ = 0.036, F-change (6, 361) = 4.711, *p* ˂ 0.001). Overall, the final full model accounted for 54.3% of the total variance in global QoL (R^2^ = 0.543, Adjusted R^2^ = 0.511). Regression diagnostics confirmed residual normality (standardized residuals range: −2.58 to 3.07), independence of errors (Durbin-Watson = 1.515), and generally acceptable collinearity across predictor variables (VIF < 5.0 for most scales, with a maximum VIF of 5.75 for physical functioning).

In the first step, demographic and clinical variables were entered into the model. Regarding sociodemographic factors, older age was significantly and negatively associated with QoL in the unadjusted initial model (Step 1: ß = −0.19, *p* = 0.005) and remained a strong, independent negative predictor in the fully adjusted model (Step 3: ß = −0.21, *p* < 0.001). Conversely, on early steps, higher education showed an initial positive association (Step 1: ß = 0.14, t = 2.80, *p* = 0.005), though its effect was fully attenuated in the final adjusted model (Step 3: ß = 0.01, *p* = 0.759). Employment status remained significantly associated with higher QoL compared to the unemployed group across white-collar (ß = 0.09, *p* = 0.044), service sector (ß = 0.20, *p* < 0.001), blue-collar occupations (ß 0.19, *p* < 0.001), and retired status (ß = 0.16, *p* = 0.022). Advanced TNM stage was strongly associated with poorer QoL in early steps (Step 1: ß = −0.18, t = −3.99, *p* < 0.001), but was attenuated in the final model (Step 3: *p* = 0.276). Gender and residency showed no statistically significant associations (*p* > 0.05). The inclusion of general functioning and symptom scales at Step 2 attenuated the associations with clinical and demographic variables. Specifically, the associations for TNM stage (ß = −0.03, *p* = 0.545) and education (ß = −0.02, *p* = 0.659) became non-significant at Step 2 and remained non-significant in the final adjusted model (Step 3: TNM stage *p* = 0.276; education ß = 0.01, *p* = 0.759). Conversely, older age maintained its strong, significant negative association across both Step 2 and the final model (Step 3: ß = −0.21, *p* < 0.001). Among the generic QoL scales at Step 2, emotional functioning displayed a significant positive association (EF: ß = 0.24, *p* < 0.001), while physical pain (PA: ß = −0.41, *p* < 0.001) and nausea/vomiting (NV: ß = −0.15, *p* = 0.016) emerged as key negative correlates.

In the final step (Step 3), disease-specific scales were added to the model. Among functional domains, emotional functioning displayed the strongest positive association with overall QoL (ß = 0.32, t = 4.78, *p* < 0.001), alongside role functioning (ß = 0.14, t = 2.23, *p* = 0.026), whereas physical functioning (ß = −0.20, t = −2.31, *p* = 0.022) and cognitive functioning (ß = −0.14, t = −2.38, *p* = 0.018) exhibited negative associations. Physical pain (ß = −0.37, t = −5.24, *p* < 0.001) and body image distress (ß = −0.12, t = −2.12, *p* = 0.035) remained persistent negative correlates. Although bivariate correlation analysis demonstrated a negative correlation between anxiety and QoL (r = −0.24, *p* < 0.001), anxiety exhibited a positive coefficient in the final model (ß = 0.24, t = 4.49, *p* < 0.001), representing a statistical suppression effect resulting from multivariable adjustment and covariance with emotional functioning and pain. The effect of nausea/vomiting was attenuated in Step 3 and reached borderline significance (ß = −0.12, t = −1.93, *p* = 0.054), whereas other gastric-specific symptom scales (such as dysphagia, reflux, eating restrictions, and hair loss) showed no statistically significant associations (*p* > 0.05).

In the fully adjusted model (Step 3, Table 4), employment status maintained a significant positive association with global health status/QoL compared to the unemployed baseline: service sector workers (B = 16.59, ß = 0.20, *p* < 0.001), blue-collar workers (B = 17.42, ß = 0.19, *p* < 0.001), retired patients (B = 7.60, ß = 0.16, *p* = 0.022), and white-collar employees (B = 7.56, ß = 0.09, *p* = 0.044). Age continuously served as a significant negative correlate (B = −0.49, ß = −0.21, *p* < 0.001). Conversely, the initial association between TNM stage and QoL observed in Step 1 (B = −6.35, *p* < 0.001) was fully attenuated in Step 3 (B = −1.60, ß = −0.05, *p* = 0.276). Similarly, education showed no significant association in the final model (B = 0.69, ß= 0.01, *p* = 0.759). These findings indicate that the relationship between disease stage and HRQoL is largely accounted for by physical and emotional symptom burdens.

The hierarchical linear regression revealed that Step 1 accounted for 22.9% of the variance (R^2^ = 0.229, Adj.R^2^ = 0.210, F (9, 377) = 12.415, *p* < 0.001). The addition of variables in Step 2 significantly improved the model (ΔR^2^ = 0.279, ΔF (10, 367) = 20.759, *p* < 0.001, Adj.R^2^ = 0.482). The final model at Step 3 explained 54.3% of the total variance (R^2^ = 0.543, ΔR^2^ = 0.036, ΔF (6, 361) = 4.711, *p* < 0.001, Adj.R^2^ = 0.511).

## 4. Discussion

This study presents data from the EORTC QLQ-C30 and the EORTC QLQ-STO22 module. This study provides important insights into health-related quality of life among gastric cancer patients in North-Eastern Kazakhstan.

Health-related quality of life (HRQoL) is increasingly recognized as a key outcome measure in both clinical trials and routine practice. Patient-reported outcome measures have already been utilized in numerous studies to evaluate HRQoL among individuals with esophageal, gastric, and gastroesophageal junction cancers [20,21,22,23,24].

One review of the current evidence summarized the impact of gastric cancer treatment on patients’ quality of life. According to the review, quality of life is one of the key considerations in selecting treatment strategies for patients with gastric cancer. When discussing available treatment options, physicians should inform patients not only about the expected survival benefits but also about the potential treatment-related side effects and their impact on quality of life, thereby enabling patients to make informed decisions and provide informed consent [21].

According to a systematic review and comparative analysis, the EORTC QLQ-C30 Summary Score is more sensitive than the Global Health Status/QoL scale in detecting differences in health-related quality of life (HRQoL). A notable statistical observation in our multivariable analysis was the elevated collinearity (VIF > 5.0 for Physical Functioning) and sign reversal of certain independent variables in Step 3. As highlighted in methodological literature [25], this pattern naturally arises because Global Health Status/QoL is derived from the same underlying instrument (EORTC QLQ-C30) as the functional and symptom subscales used to predict it. While using the QLQ-C30 Summary Score completely avoids this internal redundancy, retaining Global Health Status/QoL in our primary model allowed us to quantify the specific incremental contribution (ΔR^2^) of disease-specific symptoms (EORTC QLQ-STO22) relative to general functional perception.

Ukrainian researchers recommend assessing the quality of life of patients with gastric cancer using the EORTC questionnaires, which are considered among the most effective assessment tools. According to their study, conducted in Ukraine during the pre-war period (November 2021 to February 2022), the highest scores on the functional scales of the EORTC QLQ-C30 were observed for emotional and social functioning. On the symptom scales of the QLQ-C30, patients reported the greatest burden from financial difficulties and fatigue. The highest scores on the EORTC QLQ-STO22 were recorded for anxiety and hair loss [26].

According to a nationwide Korean study, gastric cancer survivors had poorer health-related quality of life than the general population. HRQoL was strongly associated with activity limitations, subjective health status, and sociodemographic factors, highlighting the importance of reducing physical limitations and maintaining a positive perception of health to improve patients’ quality of life [27].

Worldwide, gastric cancer remains exceptionally rare among individuals under the age of 50. The incidence rate rises progressively with age, reaching a plateau between 55 and 80 years. Additionally, men are affected at rates two to three times higher than women [28]. According to another study, young gastric cancer survivors demonstrated better physical functioning and body image than older patients; however, poorer quality of life associated with diarrhea persisted for up to one year after surgery. These findings highlight the importance of postoperative dietary monitoring and nutritional support, particularly in younger patients [29]. The findings of one study suggest that age and sex should be considered when assessing the quality of life of patients with gastric cancer [30].

The baseline characteristics of our study population (*n* = 387)—specifically the male predominance (66.7%) and the clustering of patients in the older age brackets (69.0% aged ≥ 60 years)—are representative of real-world patient demographics reported in global epidemiological studies. Age was significantly and negatively associated with QoL in the initial unadjusted model (Step 1: ß = −0.19, *p* = 0.005) and remained a strong, independent negative correlate in the fully adjusted model (Step 3: ß = −0.21, *p* < 0.001), indicating that older patients consistently reported lower quality of life. Gender and residency showed no statistically significant associations (*p* > 0.05).

Systematic review found that patient education improves overall health status as well as physical, role, emotional, cognitive, and social functioning in patients with gastric cancer following surgery, thereby significantly enhancing their quality of life [31]. According to a Vietnamese study, the lowest scores were observed in the sexual activity domain among Vietnamese patients with gastric cancer. The same study also found that poorer health-related quality of life (HRQoL) was associated with a lower educational level, older age, more advanced disease stage, and a longer time since diagnosis [32]. Although education showed an initial association with quality of life in earlier steps, its effect was fully attenuated in the final adjusted model (Step 3: ß = 0.01, *p* = 0.759). Furthermore, being employed across various occupational categories remained a consistent, positive independent predictor of overall QoL compared to the unemployed reference group: white-collar workers (ß = 0.09, *p* = 0.044), service sector workers (ß = 0.20, *p* < 0.001), and blue-collar workers (ß = 0.19, *p* < 0.001). Retired status also maintained a significant positive association (ß = 0.16, *p* = 0.022).

Choi, J. et al. (2022) showed that gastric cancer-specific symptoms, spiritual well-being, self-efficacy, and functional status affect the QoL of gastric cancer survivors [33].

According to another study, poorer health-related quality of life was associated with the lack of health insurance, a family history of cancer, and the receipt of surgical treatment alone [34].

Another study demonstrated that trait emotional intelligence influences the quality of life of patients with gastric cancer both directly and indirectly through anxiety and depression, highlighting the importance of assessing psychological factors to improve patients’ quality of life [35].

A notable statistical observation in our multivariable analysis was the sign reversal of several associated factors when moving from bivariate correlations to the final regression step. Specifically, although Anxiety (r_s_ = −0.24), Physical Functioning (PF) (r_s_ = 0.56), and Cognitive Functioning (CF) (r_s_ = 0.33) showed expected clinical relationships with global QoL in bivariate analyses, their regression coefficients inverted in Step 3 (Anxiety: B = 0.19, ß = 0.24, *p* < 0.001). Rather than reflecting a substantive clinical phenomenon—such as anxiety paradoxically enhancing quality of life—this pattern represents a classic statistical suppression effect driven by shared variance and inter-correlations among the overlapping EORTC QLQ-C30 and STO22 scales (e.g., VIF = 5.75 for PF). Instead of respecifying the comprehensive scale structure, we retained the full model to preserve the complete clinical battery, explicitly cautioning readers to interpret these inverted multivariable coefficients as statistical artifacts of multicollinearity rather than true direct clinical effects.

According to systematic review, gastric cancer resection, although clinically necessary, may have a negative impact on patients’ quality of life [36].

Another study demonstrated that differences in quality of life between long-term survivors after curative surgery for gastric cancer and the healthy population persist even five years after surgery, particularly with respect to upper gastrointestinal symptoms, including nausea and vomiting, reflux, and eating restrictions. Furthermore, despite being considered disease-free, the quality of life of patients who underwent distal subtotal gastrectomy remains affected by the long-term consequences of surgery [37].

Chinese study found that perioperative and postoperative chemotherapy had only a minimal impact on health-related quality of life (HRQoL) in patients with locally advanced gastric cancer after gastrectomy. However, perioperative chemotherapy was associated with earlier recovery of social functioning, taste perception, and anxiety [38].

According to another study, gastrectomy was associated with a temporary decline in health-related quality of life (HRQoL), with recovery occurring within approximately six months after surgery. Although patients with locally advanced gastric cancer had poorer baseline HRQoL than those with early-stage disease, these differences disappeared within three months after surgery. Furthermore, the extent of gastric resection was identified as the strongest correlate of long-term HRQoL [39].

Another study demonstrated that laparoscopic gastrectomy combined with sentinel node navigation surgery (SNNS) was associated with better postoperative quality of life than conventional laparoscopic distal gastrectomy in patients with stage I gastric cancer [40].

In a Swedish cross-sectional study of patients with newly diagnosed oesophageal and gastric cancer, the disease was found to have a profound negative impact on patients at the time of diagnosis, resulting in reduced quality of life, impaired functional status, and a high burden of symptoms [41].

A notable limitation of this study is the unmeasured nature of specific clinical variables, including exact treatment modalities (e.g., surgical procedures, ongoing versus completed chemotherapy or radiotherapy regimens), time since diagnosis, performance status, and comorbidity burden. Although gastric cancer care in Kazakhstan is clinically structured around national guidelines that align with international standards, individual treatment details were not prospectively collected to verify uniform delivery across the cohort. Nonetheless, our findings demonstrated that an advanced TNM stage was strongly associated with poorer QoL in early regression modeling (ß = −0.18, t = −3.99, *p* < 0.001), underscoring the substantial physical and psycho-emotional toll of tumor progression.

In our study, the EORTC QLQ-C30 and EORTC QLQ-STO22 multi-item scales demonstrated high internal consistency and acceptable feasibility within the North-Eastern Kazakhstani population. These findings confirm that both instruments are widely accepted and internationally standardized tools for assessing health-related quality of life in this patient cohort. Furthermore, all multi-item scales demonstrated satisfactory internal consistency (Cronbach’s α > 0.70), consistent with recent evidence on the psychometric performance of the EORTC quality-of-life instruments in patients with gastric cancer [42,43,44,45,46]. Regarding score distributions, the observed floor effects on symptom scales (e.g., Taste Problems) reflect a clinically expected absence or resolution of specific symptoms in a major subset of patients, rather than a restriction in tool measurement sensitivity. Conversely, the high ceiling effect for Social Functioning suggests a potential measurement limitation in capturing subtle variations in high-functioning individuals.

Furthermore, the positive standardized coefficient observed for anxiety in the final multivariable regression model (ß = 0.24, *p* < 0.001) must be interpreted strictly as a statistical suppression artifact rather than a beneficial clinical outcome. Bivariate correlation confirmed a significant negative association between anxiety and overall QoL (r_s_ = −0.24, *p* < 0.001). In multivariable adjustment, partialing out shared variance with collinear scales—most notably Emotional Functioning and Pain—isolated a residual covariance component that inverted the sign. Clinically, higher anxiety remains a clear indicator of psychological distress and impaired quality of life.

Rather than prescribing broad national health-policy re-organizations or mandatory popula-tion-based screening programs, these cross-sectional findings underscore the immediate clinical value of integrating routine symptom screening into outpatient oncology care. Given that pain and body image distress represent the strongest correlates of impaired quality of life in fully adjusted models, regional cancer services should prioritize tailored supportive management, early palliative symptom control, and psychosocial interventions for high-risk patients.

Several limitations of this study must be acknowledged. First, the cross-sectional design limits our ability to establish temporal causal relationships; prospective longitudinal studies are required to track Quality of Life (QoL) dynamics across different phases of the treatment trajectory. Second, our survey frame did not prospectively capture individual-level clinical details, such as specific treatment modalities (e.g., surgical extent, chemotherapy regimens), time since diagnosis, baseline performance status (ECOG), comorbidity burden, or nutritional metrics. Although gastric cancer management in Kazakhstan is structured around standardized national protocols aligned with international guidelines (such as ESMO/NCCN), the assumption that our cohort reflects a uniform standard-of-care distribution should be interpreted with caution due to unmeasured treatment variability and potential residual confounding. Third, potential selection bias is inherent to our consecutive convenience sampling methodology. The exclusion of patients with severe comorbidities, end-stage disease, or cognitive impairment created a survival filter, leading to an overrepresentation of early-to-intermediate stage disease (61.0% Stage II) and a low proportion of Stage IV patients (5.4%). Consequently, our cohort primarily reflects stable, ambulatory patients, which may underestimate the true symptom burden in unselected or late-stage clinical populations. Data on the number of patients approached or declining participation prior to consent were not systematically recorded during the consecutive convenience sampling process; tracking began only from the point of written informed consent and questionnaire distribution (*n* = 401 collected), precluding calculation of a pre-consent response rate and construction of a formal STROBE flow diagram (Items 13–14). Data quality exclusions applied after collection (*n* = 14, due to >50% missing items per scale) are reported separately in Section 2.1. This gap in pre-consent recruitment tracking is acknowledged as a limitation. Fourthly, multicollinearity among closely linked psychometric scales (such as Physical Functioning, Role Functioning, and Pain, with VIFs reaching 5.75) presents an inherent feature of comprehensive health-related quality of life instruments. While overall model fit (R^2^ = 0.543) remained robust, individual regression coefficients should be interpreted with caution due to variance sharing among collinear subscales. Finally, data were gathered exclusively from North-Eastern Kazakhstan, necessitating multi-center national studies to verify broader generalizability. Additionally, formal psychometric validation of the EORTC QLQ-C30 and QLQ-STO22 instruments in this population—including confirmatory factor analysis, test–retest reliability, and measurement invariance testing between the Russian-completed and Kazakh-assisted subgroups—was beyond the scope of this study; our findings are limited to internal consistency (Cronbach’s α) and feasibility (floor/ceiling) metrics.

Despite these limitations, a major strength of this study is the rigorous evaluation of two internationally recognized patient-reported outcome measures (EORTC QLQ-C30 and QLQ-STO22) in North-Eastern Kazakhstan, establishing a methodologically structured approach for future oncological survivorship research in the region.

## 5. Conclusions

In conclusion, this cross-sectional study evaluated factors associated with health-related quality of life (HRQoL) among gastric cancer patients in North-Eastern Kazakhstan. Both the EORTC QLQ-C30 and QLQ-STO22 instruments displayed high internal reliability and feasibility, supporting their utility for patient-reported outcome measurement in this clinical setting. In initial multivariable models, advanced TNM stage and higher education were associated with HRQoL outcomes; however, these associations were substantially attenuated after adjusting for physical and emotional symptom burden in fully adjusted models (*p* = 0.276 for TNM stage and *p* = 0.759 for education). Older age and physical symptoms—particularly pain and body image disturbances—remained the strongest independent factors associated with HRQoL deficits. Rather than prescribing structural service reorganizations, these findings highlight the importance of routine symptom screening, tailored supportive care, and psychosocial management in regional oncology practice.

## Figures and Tables

**Table 1:** Sociodemographic characteristics of the sample.

		*N*	%
Gender			
	Male	258	66.7
	Female	129	33.3
Age			
	Below 50 years	42	10.8
	51–60 years	78	20.2
	61–70 years	153	39.5
	Above 71 years	114	29.5
Residency			
	Urban	316	81.7
	Rural	71	18.3
Education			
	Higher	123	31.8
	Secondary	264	68.2
Employment			
	White-collar workers	38	9.8
	Service sector workers	38	9.8
	Blue-collar workers	31	8.0
	Unemployed	74	19.1
	Retired	206	53.2
TNM stages			
	I	36	9.3
	II	236	61.0
	III	94	24.3
	IV	21	5.4

**Table 2:** Mean ± SD Floor *n* (%), Ceiling *n* (%), and Cronbach’s Alpha Values for EORTC QLQ-C30 and QLQ-STO22 Scales.

Items (Items Numbers)	Missing *n* (%)	Mean ± SD *	Floor *n* (%)	Ceiling *n* (%)	Cronbach’s
EORTC QLQ-C30 **					
Global health status/QoL (QL)	0 (0)	51.9 ±24.3	6 (1.6)	6 (1.6)	0.94
Physical functioning (PF)	0 (0)	68.0 ±26.2	2 (0.5)	24 (6.2)	0.92
Role Functioning (RF)	0 (0)	71.0 ±27.7	13 (3.4)	134 (34.6)	0.88
Emotional functioning (EF)	0 (0)	62.6 ± 27.4	6 (1.6)	48 (12.4)	0.90
Cognitive functioning (CF)	0 (0)	69.5 ± 22.4	2 (0.5)	79 (20.4)	0.70
Social functioning (SF)	0 (0)	78.9 ± 24.1	10 (2.6)	158 (40.8)	0.75
Fatigue (FA)	0 (0)	44.9 ± 23.8	19 (4.9)	4 (1.0)	0.80
Nausea and vomiting (NV)	0 (0)	20.1 ± 24.8	198 (51.2)	2 (0.5)	0.84
Pain (PA)	0 (0)	30.7 ± 27.9	124 (32.0)	10 (2.6)	0.83
Dyspnea (DY)	0 (0)	27.5 ± 32.5	200 (51.7)	24 (6.2)	
Insomnia (SL)	0 (0)	42.1 ± 31.0	93 (24.0)	36 (9.3)	
Appetite loss (AP)	0 (0)	40.6 ± 29.1	92 (23.8)	22 (5.7)	
Constipation (CO)	0 (0)	19.3 ± 23.8	210 (54.3)	4 (1.0)	
Diarrhea (DI)	0 (0)	23.9 ± 24.9	170 (43.9)	8 (2.1)	
Financial difficulties (FI)	0 (0)	35.7 ± 36.3	170 (43.9)	46 (11.9)	
QLQ-STO22 ***					
Dysphagia	0 (0)	25.5 ± 19.9	69 (17.8)	2 (0.5)	0.72
Stomach area pain	0 (0)	28.4 ± 21.4	55 (14.2)	2 (0.5)	0.78
Reflux	0 (0)	28.6 ± 19.3	33 (8.5)	4 (1.0)	0.71
Eating restrictions	0 (0)	27.0 ± 20.4	65 (16.8)	4 (1.0)	0.71
Anxiety	0 (0)	61.1 ± 30.5	26 (6.7)	82 (21.2)	0.74
Dry mouth	0 (0)	25.7 ± 26.7	172 (44.4)	6 (1.6)	
Taste problem	0 (0)	18.5 ± 26.2	234 (60.5)	10 (2.6)	
Body image	0 (0)	36.6 ± 23.8	53 (13.7)	11 (2.8)	
Hair loss	0 (0)	25.8 ± 26.4	154 (39.8)	13 (3.4)	

* SD = Standard deviations, ** Scores range from 0 to 100 with higher scores indicating better conditions, *** Scores range from 0 to 100 with higher scores indicating greater level of symptoms.

**Table 3:** Spearman’s correlation matrix between EORTC QLQ-C30 and QLQ-STO22 scales and Global Health Status.

	GHS	PF	RF	EF	CF	SF	FA	PA	DY	NV	AP	SL	CO	DI	FI	DYS	ER	Reflux	Anxiety	BI	Hair Loss
GHS	1																				
PF	0.56	1																			
RF	0.51	0.80	1																		
EF	0.51	0.67	0.51	1																	
CF	0.33	0.63	0.51	0.69	1																
SF	0.48	0.68	0.60	0.61	0.54	1															
FA	−0.45	−0.67	−0.63	−0.52	−0.56	−0.54	1														
PA	−0.61	−0.81	−0.74	−0.57	−0.51	−0.64	0.68	1													
DY	−0.38	−0.66	−0.50	−0.53	−0.44	−0.40	0.42	0.52	1												
NV	−0.55	−0.71	−0.59	−0.61	−0.59	−0.61	0.55	0.71	0.48	1											
AP	−0.29	−0.44	−0.37	−0.36	−0.39	−0.32	0.57	0.37	0.39	0.43	1										
SL	−0.35	−0.53	−0.48	−0.58	−0.51	−0.42	0.62	0.51	0.43	0.44	0.43	1									
CO	−0.40	−0.46	−0.38	−0.37	−0.31	−0.46	0.27	0.47	0.39	0.57	0.22	0.24	1								
DI	−0.32	−0.44	−0.42	−0.47	−0.46	−0.34	0.38	0.39	0.32	0.46	0.40	0.40	0.22	1							
FI	−0.36	0.22	0.10	0.08	−0.06	0.02	0.04	−0.23	−0.40	−0.16	0.05	0.02	−0.22	0.05	1						
DYS	−0.38	−0.66	−0.50	−0.53	−0.44	−0.40	0.42	0.52	0.47	0.48	0.39	0.43	0.42	0.43	0.05	1					
ER	−0.43	−0.59	−0.50	−0.54	−0.56	−0.50	0.51	0.54	0.39	0.52	0.30	0.43	0.26	0.43	0.10	0.39	1				
Reflux	−0.42	−0.59	−0.38	−0.60	−0.50	−0.40	0.44	0.53	0.50	0.55	0.32	0.38	0.38	0.30	−0.27	0.50	0.40	1			
Anxiety	−0.24	−0.41	−0.32	−0.59	−0.43	−0.34	0.43	0.36	0.35	0.38	0.26	0.41	0.20	0.23	−0.04	0.35	0.30	0.44	1		
BI	−0.29	−0.50	−0.45	−0.47	−0.49	−0.45	0.50	0.44	0.22	0.45	0.29	0.36	0.24	0.32	0.21	0.22	0.54	0.33	0.59	1	
Hair loss	−0.13	−0.38	−0.26	−0.34	−0.33	−0.19	0.28	0.28	0.29	0.33	0.20	0.24	0.14	0.16	0.00	0.28	0.28	0.36	0.31	0.33	1

Note: Values are Spearman’s correlation coefficients (r_s_). All presented interscale correlations are statistically significant at *p* < 0.05 (two-tailed). GHS—Global Health Status/Quality of Life; PF—Physical Functioning; RF—Role Functioning; EF—Emotional Functioning; CF—Cognitive Functioning; SF—Social Functioning; FA—Fatigue; PA—Pain; DY—Dyspnea; NV—Nausea and Vomiting; AP—Appetite Loss; SL—Insomnia (Sleep Disturbance); CO—Constipation; DI—Diarrhea; FI—Financial difficulties; DYS—Dysphagia; ER—Eating Restrictions; Reflux—Reflux Symptoms; Anxiety—Anxiety; BI—Body Image; Hair loss—Hair Loss; EORTC QLQ-C30—European Organization for Research and Treatment of Cancer QOL measure.

**Table 4:** Hierarchical Multiple Regression Analysis Predicting Global Health Status/QoL (QLQ-C30, QLQ-STO22).

Variable	B	95%CI	SE	ß	t	*p*	Tolerance	VIF
	Step 1: Clinical & Demographic							
Gender	3.01	[−1.81, 7.83]	2.45	0.06	1.23	0.221	0.899	1.11
Age	−0.44	[−0.74, −0.13]	0.16	−0.19	−2.81	0.005	0.452	2.21
Residency	4.23	[−1.54, 9.99]	2.93	0.07	1.44	0.150	0.933	1.07
Education	7.06	[2.10, 12.02]	2.52	0.14	2.80	0.005	0.871	1.15
Employment (Ref.: Unemployed)								
emp_1 White-collar workers	4.27	[−4.68, 13.23]	4.56	0.05	0.94	0.349	0.654	1.53
emp_2 Service sector workers	20.13	[11.48, 28.78]	4.40	0.25	4.57	0.000	0.701	1.43
emp_3 Blue-collar workers	17.23	[7.69, 26.76]	4.85	0.19	3.55	0.000	0.694	1.44
emp_5 Retired	1.29	[−6.61, 9.18]	4.02	0.03	0.32	0.748	0.299	3.34
TNM stages	−6.35	[−9.48, −3.23]	1.59	−0.18	−3.99	0.000	0.980	1.02
	Step 2: EORTC QLQ-C30 Scales							
Gender	3.54	[−0.53, 7.60]	2.07	0.07	1.71	0.088	0.829	1.21
Age	−0.49	[−0.74, −0.23]	0.13	−0.21	−3.77	0.000	0.426	2.35
Residency	2.07	[−2.79, 6.94]	2.48	0.03	0.84	0.403	0.858	1.16
Education	−0.99	[−5.41, 3.42]	2.25	−0.02	−0.44	0.659	0.721	1.39
Employment (Ref.: Unemployed)								
emp_1 White-collar workers	7.26	[−0.16, 14.69]	3.78	0.09	1.92	0.055	0.624	1.60
emp_2 Service sector workers	14.92	[7.66, 22.18]	3.69	0.18	4.04	0.000	0.653	1.53
emp_3 Blue-collar workers	17.42	[9.58, 25.26]	3.99	0.19	4.37	0.000	0.673	1.48
emp_5 Retired	6.89	[0.42, 13.36]	3.29	0.14	2.10	0.037	0.293	3.41
TNM stages	−0.89	[−3.78, 2.00]	1.47	−0.03	−0.61	0.545	0.750	1.33
PF	−0.15	[−0.30, 0.01]	0.08	−0.16	−1.91	0.057	0.186	5.37
RF	0.12	[0.02, 0.24]	0.06	0.14	2.24	0.026	0.327	3.06
EF	0.21	[0.11, 0.32]	0.06	0.24	3.89	0.000	0.346	2.89
CF	−0.12	[−0.24, 0.01]	0.06	−0.11	−1.92	0.055	0.411	2.43
SF	0.02	[−0.09, 0.13]	0.06	0.02	0.40	0.690	0.448	2.23
FA	−0.03	[−0.17, 0.10]	0.07	−0.03	−0.48	0.633	0.298	3.35
PA	−0.35	[−0.47, −0.23]	0.06	−0.41	−5.66	0.000	0.259	3.85
NV	−0.15	[−0.26, −0.03]	0.06	−0.15	−2.43	0.016	0.354	2.87
AP	−0.01	[−0.09, 0.07]	0.04	−0.01	−0.21	0.838	0.581	1.72
SL	0.07	[−0.01, 0.15]	0.04	0.09	1.71	0.089	0.490	2.04
	Step 3: EORTC QLQ-STO22 Scales							
Gender	2.07	[−1.98, 6.13]	2.06	0.04	1.00	0.316	0.786	1.27
Age	−0.49	[−0.74, −0.23]	0.13	−0.21	−3.75	0.000	0.396	2.52
Residency	1.56	[−3.30, 6.42]	2.47	0.03	0.63	0.529	0.811	1.23
Education	0.69	[−3.72, 5.10]	2.24	0.01	0.31	0.759	0.680	1.47
Employment (Ref.: Unemployed)								
emp_1 White-collar workers	7.56	[0.20, 14.93]	3.74	0.09	2.02	0.044	0.599	1.67
emp_2 Service sector workers	16.59	[9.32, 23.85]	3.69	0.20	4.50	0.000	0.616	1.62
emp_3 Blue-collar workers	17.42	[9.75, 25.08]	3.90	0.19	4.47	0.000	0.664	1.51
emp_5 Retired	7.60	[1.10, 14.09]	3.30	0.16	2.30	0.022	0.274	3.65
TNM stages	−1.60	[−4.49, 1.29]	1.47	−0.05	−1.09	0.276	0.707	1.41
PF	−0.18	[−0.34, −0.03]	0.08	−0.20	−2.29	0.022	0.174	5.75
RF	0.12	[0.01, 0.23]	0.06	0.14	2.23	0.026	0.309	3.24
EF	0.29	[0.17, 0.40]	0.06	0.32	4.78	0.000	0.276	3.62
CF	−0.15	[−0.27, −0.02]	0.06	−0.14	−2.37	0.018	0.388	2.57
SF	0.02	[−0.09, 0.13]	0.05	0.02	0.35	0.726	0.418	2.39
FA	−0.02	[−0.16, 0.11]	0.07	−0.02	−0.32	0.745	0.280	3.57
PA	−0.32	[−0.44, −0.20]	0.06	−0.37	−5.24	0.000	0.250	4.00
NV	−0.11	[−0.23, −0.00]	0.06	−0.12	−1.93	0.054	0.340	2.94
AP	−0.03	[−0.11, 0.05]	0.04	−0.03	−0.69	0.490	0.541	1.85
SL	0.05	[−0.03, 0.13]	0.04	0.07	1.27	0.205	0.470	2.13
DYS	0.08	[−0.06, 0.22]	0.07	0.06	1.09	0.278	0.368	2.72
ER	−0.12	[−0.26, 0.02]	0.07	−0.09	−1.67	0.096	0.432	2.31
Reflux	−0.09	[−0.20, 0.01]	0.05	−0.08	−1.67	0.095	0.491	2.03
Anxiety	0.19	[0.11, 0.27]	0.04	0.24	4.49	0.000	0.453	2.20
BI	−0.12	[−0.23, −0.01]	0.06	−0.12	−2.12	0.035	0.406	2.46
Hair loss	0.03	[−0.04, 0.10]	0.04	0.03	0.77	0.439	0.682	1.47
Model Step	R^2^	Adjusted R^2^	ΔR^2^	F change	df1	df2	*p*	Durbin-Watson
Step 1	0.229	0.210	0.229	12.415	9	377	<0.001	
Step 2	0.507	0.482	0.279	20.759	10	367	<0.001	
Step 3	0.543	0.511	0.079	8.885	6	361	<0.001	1.515

SE—Standard Error; VIF—Variance Inflation Factor. Note: Dyspnea, constipation, diarrhea, and financial difficulties scales were omitted from the regression analysis to prevent high multicollinearity, despite significant bivariate correlations with the outcome measure.

## Data Availability

The data presented in this study are available on reasonable request from the corresponding author due to privacy considerations.
